# Tree diversity increases productivity through enhancing structural complexity across mycorrhizal types

**DOI:** 10.1126/sciadv.adi2362

**Published:** 2023-10-06

**Authors:** Tama Ray, Benjamin M. Delory, Rémy Beugnon, Helge Bruelheide, Simone Cesarz, Nico Eisenhauer, Olga Ferlian, Julius Quosh, Goddert von Oheimb, Andreas Fichtner

**Affiliations:** ^1^ Institute of General Ecology and Environmental Protection, Technische Universität Dresden, Tharandt, Germany.; ^2^ German Centre for Integrative Biodiversity Research (iDiv) Halle-Jena-Leipzig, Leipzig, Germany.; ^3^ Institute of Biology/Geobotany and Botanical Garden, Martin Luther University Halle-Wittenberg, Halle (Saale), Germany.; ^4^ Institute of Ecology, Leuphana University of Lüneburg, Lüneburg, Germany.; ^5^ Leipzig Institute for Meteorology, Universität Leipzig, Stephanstraße 3, 04103 Leipzig, Germany.; ^6^ CEFE, Univ Montpellier, CNRS, EPHE, IRD, 1919, route de Mende, F-34293 Montpellier Cedex 5, France.; ^7^ Institute of Biology, Leipzig University, Leipzig, Germany.

## Abstract

Tree species diversity and mycorrhizal associations play a central role for forest productivity, but factors driving positive biodiversity-productivity relationships remain poorly understood. In a biodiversity experiment manipulating tree diversity and mycorrhizal associations, we examined the roles of above- and belowground processes in modulating wood productivity in young temperate tree communities and potential underlying mechanisms. We found that tree species richness, but not mycorrhizal associations, increased forest productivity by enhancing aboveground structural complexity within communities. Structurally complex communities were almost twice as productive as structurally simple stands, particularly when light interception was high. We further demonstrate that overyielding was largely explained by positive net biodiversity effects on structural complexity with functional variation in shade tolerance and taxonomic diversity being key drivers of structural complexity in mixtures. Consideration of stand structural complexity appears to be a crucial element in predicting carbon sequestration in the early successional stages of mixed-species forests.

## Teaser

Stand structural complexity drives positive biodiversity-productivity relationships in forests.

## INTRODUCTION

Numerous studies showed that forest productivity increases with tree species richness at local (*1*) and global scales (*2*). The mechanisms underlying these positive biodiversity-productivity relationships (BPRs) in forest ecosystems have been the focus of a range of tree biodiversity experiments set up in different biomes (*3*). Species interactions at the local neighborhood scale—which can lead to reduced competition through niche partitioning or facilitation (*4*), spatial complementarity of tree crowns in canopy space due to neighbor-mediated shifts in crown traits and allocation patterns (*5*, *6*), and temporal variation in functional traits within a community (*7*)—have been identified as important drivers of overyielding in experimental tree communities (i.e., higher yield in a mixture compared to the weighted average of monoculture yields) (*8*). By contrast, it remains unclear how overyielding is linked to the structural complexity (i.e., the structural diversity in three-dimensional space) of forests.

In forests, stand structural complexity is caused by a high variation in tree size and a high dissimilarity in the spatial arrangement of tree crowns. It has been quantified with several indices, often based on one- or two-dimensional stand structural attributes such as variation in tree diameter and tree height or stand density (*9*, *10*). Exploring the role of forest structural complexity in regulating species interactions and stand productivity, however, requires accurate quantification of the three-dimensional morphology of individual trees and the space occupied by growing trees (*11*). Novel approaches, such as terrestrial laser scanning (TLS), have been applied to characterize the structural complexity within a stand by summarizing information extracted from point clouds into a stand structural complexity index [SSCI; (*12*)]. This index captures different facets of the structural complexity of forest plots: (i) the vertical distribution of structural elements, such as stems, branches, and leaves and (ii) the density of these elements in three-dimensional space. Thus, SSCI encapsulates information about the three-dimensional heterogeneity in biomass distribution of all trees within a community (*13*, *14*).

The structural complexity of a stand is shaped by tree species diversity (*14*, *15*). Several studies using SSCI showed that stands become structurally more complex with increasing tree species richness (*16*–*18*) and that positive biodiversity effects on complexity become stronger over time (*16*). Stand structural complexity is strongly related to canopy space occupation. For example, differences in crown architecture among species and neighborhood-driven plasticity within species (*19*), such as plastic changes in branch traits and branching patterns (*20*), contribute to greater canopy complexity in mixed-species tree communities compared to monocultures. In particular, the physical complementarity between trees in the vertical and horizontal space of the canopy allows species-rich tree communities to exploit light resources more efficiently (*5*, *21*), with shade-tolerant and light-efficient species occupying lower canopy layers (*22*). This more efficient light use in mixtures should lead to higher aboveground productivity in structurally more complex forest stands. This positive relationship between structural complexity and forest productivity has been demonstrated in natural forests using two-dimensional structural complexity measures (*23*, *24*) or canopy rugosity (*25*). However, we still have a limited understanding of how tree species richness modulates the relationship between stand structural complexity and forest productivity, especially when structural complexity is measured using state-of-the-art techniques that allow for detailed characterization of tree biomass distribution in three-dimensional space.

While these approaches allow us to assess the role of aboveground structural complexity for productivity, next to nothing is known about how belowground biotic interactions affect this relationship. Associations between mycorrhizal fungi and tree roots are an essential type of interaction in forests (*26*–*28*). Temperate tree species are associated with ectomycorrhizal fungi (EM) belonging to Asco- or Basidiomycetes, arbuscular mycorrhizal (AM) fungi of the phylum Glomeromycota (*29*), or simultaneously with both EM and AM fungi (*30*, *31*). While EM tree species generally benefit from greater nitrogen mobilization from organic matter and enhanced organic and inorganic resource uptake (*32*), trees associated with AM fungi mainly benefit from greater uptake of less mobile nutrients such as phosphorus (*26*–*29*). Mycorrhizal associations with AM or EM fungi are known to influence tree productivity differently (*33*–*35*). For instance, Deng *et al.* (*36*) found that differences in nutrient acquisition strategies affect the direction of BPRs, with positive effects of tree species richness on AM tree productivity but negative effects for EM trees. Despite the importance of mycorrhizal associations in mediating tree productivity, the extent to which functionally distinct mycorrhizal associations influence the structural complexity of forest stands remains to be elucidated.

In this study, we took advantage of a tree diversity experiment called MyDiv. Established in 2015, the MyDiv experiment is located in eastern Germany and was designed to explore the role of mycorrhizal associations in shaping biodiversity-ecosystem functioning relationships in temperate tree communities (*37*). This unique experiment consists of two orthogonal gradients of tree species richness and mycorrhizal associations, where communities were planted with tree species that preferentially associate with arbuscular mycorrhiza (AM trees), tree species that preferentially associate with ectomycorrhiza [EM trees; (*31*)], or a combination of the two mycorrhizal types (50% AM and 50% EM trees) along a gradient of tree species richness (monocultures, two-species mixtures, and four-species mixtures). Here, we aimed to disentangle the relative importance of stand structural complexity and mycorrhizal associations in modulating BPRs in young temperate tree communities. First, we tested the hypothesis that the structural complexity and wood productivity of tree communities increase with tree species richness and that mixtures that have both, AM and EM tree species, are most productive and structurally more complex. In addition, we investigated whether light interception modulates complexity-productivity relationships in young forest stands. Second, we determined pathways by which tree species richness affects community productivity. We hypothesized that tree species richness increases productivity by enhancing communities’ structural complexity and by decreasing tree mortality within a community, a further important factor for regulating productivity. Furthermore, we expected that a lower proportion of AM and EM trees in a community, which occurs when switching from monocultures of AM or EM trees (100%) to mixed communities (50% AM and 50% EM trees), would have an additional positive effect on wood productivity. Third, we explored links between tree species richness, structural complexity, overyielding, and functional characteristics of tree communities. We focused our analysis on two functional characteristics of tree communities: the community-weighted mean (CWM) and the functional dispersion (FD) of shade tolerance. We hypothesized that the positive net effect of tree species richness on stand structural complexity is largely driven by differences in shade tolerance among tree species within a community. In particular, we expected biodiversity effects on stand structural complexity to be greater in species-rich tree communities composed of species that were less similar in their shade tolerance. To test these hypotheses, we measured stand structural complexity [SSCI, sensu (*12*)] in 2021 using TLS and quantified annual wood productivity (AWP) (increment in stem volume from 2015 to 2021) by measuring the radial and longitudinal growth of individual trees. For each tree species included in our experiment, shade tolerance values were taken from the literature (*38*).

## RESULTS

### Stand structural complexity and productivity increase with tree species richness but are not affected by mycorrhizal associations

**Fig. 1.**
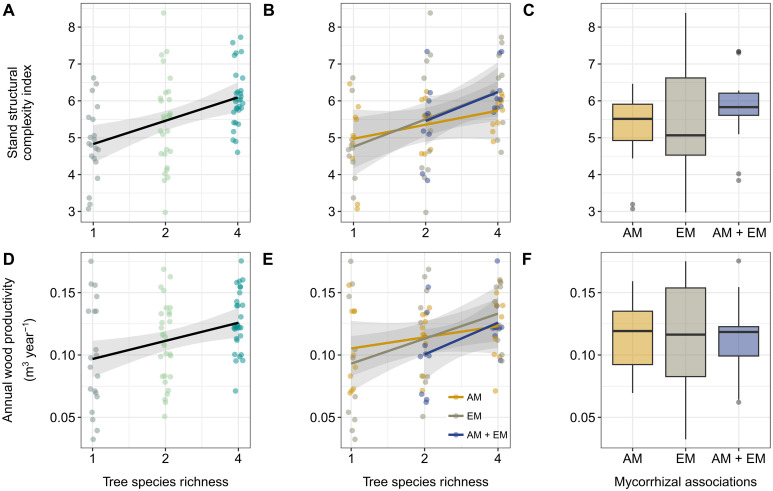
Effects of tree species richness and mycorrhizal associations on stand structural complexity and community productivity. (**A** and **D**) show variations in SSCI and AWP as affected by tree species richness across mycorrhizal associations (SSCI: *P* = 0.005, *R*^2^ = 0.19; AWP: *P* = 0.006, *R*^2^ = 0.11), while (**B** and **E**) show these relationships for each mycorrhizal association separately. The *R*² value indicates the proportion of variance explained by SR alone. Note that the interaction between tree species richness and mycorrhizal association was not statistically significant (SSCI: *P* = 0.67; AWP: *P* = 0.54). The solid lines are mixed-effect model fits, and the shaded areas indicate the 95% confidence interval of the prediction. Individual dots represent the observed SSCI or AWP values, which are jittered to improve readability. (**C** and **F**) show how SSCI and AWP were affected by mycorrhizal associations (across tree species richness levels). Boxplots show the median (horizontal black lines), the 25 and 75% percentiles (edges of the box), and 1.5 times the interquartile range (whiskers) of observed SSCI or AWP values. Gray dots indicate SSCI or AWP values that are greater or smaller than 1.5 times the interquartile range. Differences among mycorrhizal associations were not statistically significant (Tukey’s test: *P* > 0.10).

SSCI increased with tree species richness (*P* = 0.005) and was, on average, 25% higher in four-species mixtures compared to monocultures (Fig. 1A). The magnitude and direction of this relationship, however, were consistent across mycorrhizal associations (Table 1 and Fig. 1B), and SSCI did not significantly vary among mycorrhizal associations either (Table 1 and Fig. 1C). Across mixtures, the magnitude of tree species richness effects was strong (Hedges’ *g*: 0.95), and effect sizes were almost three times higher in four-species than in two-species mixtures (Hedges’ *g*: 1.49 and 0.57, respectively; fig. S1).

**Table 1.** Effects of tree species richness and mycorrhizal associations on stand structural complexity (SSCI) and community productivity (AWP). Results of linear mixed-effect models using type III sum of squares. Tree species richness (log_2_SR) was fitted as a numeric variable, with mycorrhizal association being a categorical variable with three levels (AM, EM, and AM + EM). AWP, annual wood productivity; LI, mean light intensity at ground level; df, numerator degrees of freedom; ddf, denominator degrees of freedom. See table S1 for information on random effects and model fits of the best-fitted model. Because light intensity data were only available for half of the plots, the mixed-effect model linking AWP, SSCI, and light intensity was fitted on half of the data. *P* < 0.05 are highlighted in bold.

	df	ddf	*F* value	*P* value
**SSCI**				
Species richness (SR)	1	52.7	8.41	**0.005**
Mycorrhizal association (MA)	2	52.8	0.10	0.903
SR x MA	2	52.0	0.40	0.671
				
**AWP**				
Species richness (SR)	1	54.7	8.11	**0.006**
Mycorrhizal association (MA)	2	53.1	0.80	0.453
SR x MA	2	50.6	0.63	0.535
				
**AWP** (subsetted data)				
Stand structural complexity (SSCI)	1	33.4	19.75	**0.000**
Mean light intensity at ground level (LI)	1	34.6	5.17	**0.029**
SSCI x LI	1	34.6	7.88	**0.008**

Overall, the AWP of tree communities increased with tree species richness (*P* = 0.006; Fig. 1D). After 6 years, four-species mixtures accumulated an average of 29% more wood volume than monocultures. However, we found no support for the hypothesis that the relationship between tree species richness and AWP was modulated by mycorrhizal associations within tree communities (Table 1) despite the fact that communities with both AM and EM tree species exhibited the strongest increase in AWP with tree species richness (Fig. 1E). AM communities shifted from being the most productive to being the least productive along the diversity gradient. In contrast, EM communities benefited most from growing in four-species mixtures (fig. S2), although these trends were not statistically significant (*P* > 0.05). Across tree communities, AWP did not differ significantly among mycorrhizal associations (all comparisons *P*_adj._ > 0.10), with variability in AWP being largest for EM communities (coefficient of variation: AM = 0.24, EM = 0.38; AM + EM = 0.25; Fig. 1F).

### Tree productivity increases with stand structural complexity, but the strength of this relationship is modulated by light conditions

**Fig. 2.**
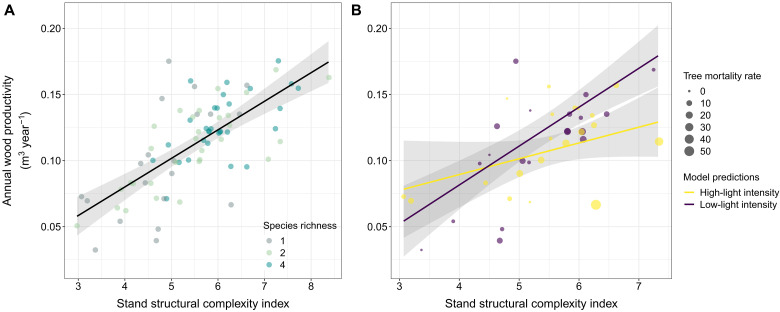
Relationship between stand structural complexity and community productivity. (**A**) shows changes in AWP with SSCI across tree communities (monocultures and mixtures). (**B**) shows how mean light intensity at ground level (used as a proxy for light interception) modulates the effects of SSCI on AWP. Regression lines correspond to changes in AWP in response to SSCI at low and high values of light intensity (computed as the 25 and 75% quantile of light intensity). The size of the points corresponds to the total tree mortality rate (%) within a stand during the census interval (2015–2021), while the color of the points indicates stands that exhibited lower (yellow) and higher (purple) light intensity values than the median. The solid lines in (A) and (B) are mixed-effect model fits, with shaded areas indicating the 95% confidence interval of the prediction. Points represent AWP and SSCI values measured in each plot.

Both monocultures and mixtures became more productive with increasing stand structural complexity (*P <* 0.001; Fig. 2A). For example, the AWP of communities with a high structural complexity (95% quantile of SSCI = 7.3) was almost twofold (+88%) higher than those associated with a low structural complexity (5% quantile of SSCI = 3.8).

To test the importance of stand-level light interception in mediating the positive relationship between stand structural complexity and productivity, we calculated the average light intensity at ground level (20 cm) during the day in half of our study plots and used it as a proxy for light interception by stand structural elements (i.e., the lower the ground light intensity, the greater the light interception). Across the tree species richness gradient, we found that the strength of the positive complexity-productivity relationship was dependent on ground light intensity (interaction between SSCI and light intensity: *P* = 0.008; Table 1). At high levels of ground light intensity (i.e., low-light interception), changes in SSCI did not result in significant changes in AWP (slope of the AWP-SSCI relationship: 0.012; confidence interval (CI): [−0.002, 0.026]; *P* = 0.099; Fig. 2B). In contrast, AWP strongly increased with SSCI in communities where ground light intensity was low (i.e., high-light interception, slope of the AWP-SSCI relationship: 0.029; CI: [0.019, 0.040]; *P* < 0.0001; Fig. 2B). Because of sampling issues, this analysis was limited to a subset of plots that did not cover the entire range of observed SSCI values and excluded the most structurally complex stands. However, we assume that the observed pattern would become even stronger if the plots with the greatest SSCI values were also taken into account. On average, tree mortality, which can affect light interception by providing gaps in tree canopies, was not different in stands with high-light (i.e., greater than the median) or low-light (i.e., lower than the median) intensity at ground level (*P* = 0.42; fig. S3).

### The positive effect of tree species richness on wood productivity is mediated by stand structural complexity

**Fig. 3.**
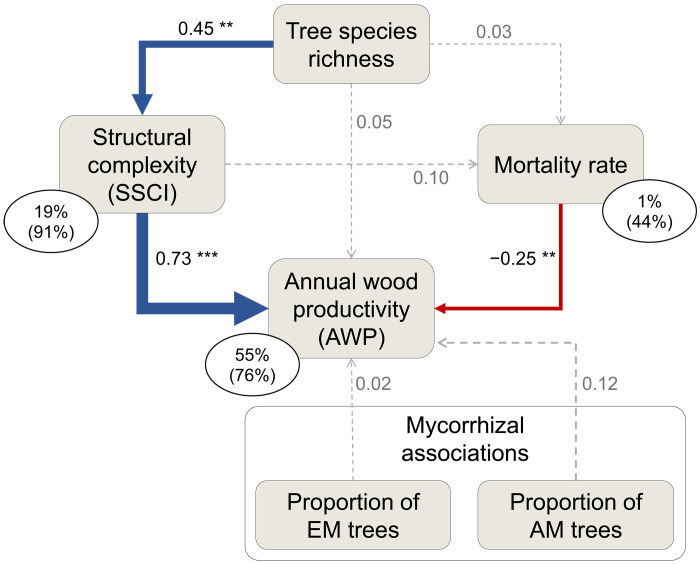
Structural equation model relating tree species richness, structural complexity, mortality rate, and mycorrhizal associations to AWP of tree communities. The blue and red arrows indicate significant (*P < 0.05, **P < 0.01, and ***P < 0.001) positive and negative relationships, respectively. Arrow width is proportional to standardized path coefficients. Dotted gray arrows indicate nonsignificant (*P* > 0.10) paths. Numbers next to arrows are standardized path coefficients. Percentage values are proportions of variance explained by fixed effects; proportions of variance explained by fixed and random effects are in parentheses. Note that communities with both AM and EM tree species were coded as a reference in the SEM. This SEM fitted our data well (Fisher’s *C* = 9.41, df = 8, *P* = 0.309).

To understand how tree species richness and mycorrhizal associations affected tree mortality, structural complexity (SSCI), and wood productivity (AWP) in our young temperate forest plots, we fitted a first structural equation model (SEM) to our data (Fig. 3). Tree species richness, SSCI, tree mortality, and mycorrhizal associations (i.e., proportion of AM and EM trees in communities) explained 55% of the variation in AWP (Fig. 3). We found that the positive effect of tree species richness on AWP was mediated by SSCI. SSCI was the strongest driver of AWP. Even after controlling for other fixed effects in the productivity model and the influence of differences in tree species composition between communities, SSCI still explained a substantial amount (47.5%) of the variation in AWP. As expected, tree mortality had a negative effect on AWP but was not related to SSCI or tree species richness. The relative importance of direct effects of tree mortality rate (*R*^2^ = 5.5%), tree species richness (*R*^2^ = 0.2%), and mycorrhizal associations (AM tree communities, *R*^2^ = 1.2%; EM tree communities, *R*^2^ = 0.03%) on AWP was small. We only found weak nonsignificant relationships between the proportion of AM and EM tree species in communities and AWP (Fig. 3).

### Tree species richness and interspecific variation in shade tolerance simultaneously drive stand structural complexity in mixtures, resulting in stronger biodiversity effects on wood productivity in structurally more complex stands

**Fig. 4.**
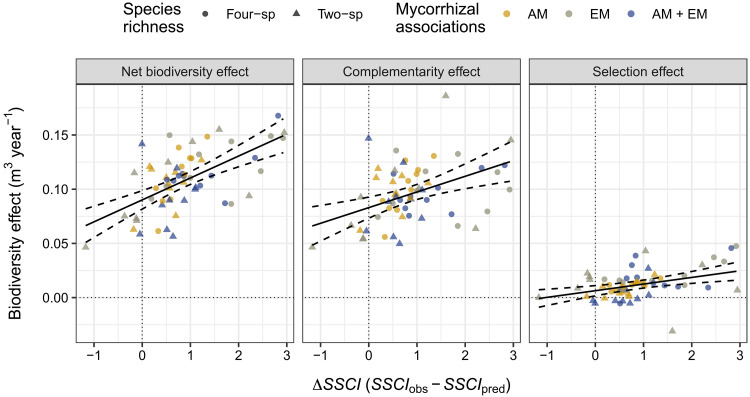
Overyielding in mixed-species communities is driven by stronger complementarity effects in structurally more complex tree communities. Points are plot-level estimates of biodiversity effects on wood productivity and stand structural complexity (Δ*SSCI*). Solid black lines are predictions from mixed-effect models. Dashed lines indicate the 95% confidence interval of the prediction. Δ*SSCI* explains 35.6% (*P* < 0.0001), 18.0% (*P* = 0.0009), and 13.0% (*P* = 0.0016) of the variation in NBEs, complementarity effects, and selection effects, respectively.

For each mixture plot, we quantified the net effect of tree species richness on (i) wood productivity (i.e., overyielding) and (ii) stand structural complexity (Δ*SSCI*). The net biodiversity effect (NBE) on wood productivity was calculated and partitioned into complementarity and selection effects following (*8*). The net effect of tree species richness on stand structural complexity was estimated by calculating the difference between the SSCI measured by TLS in mixed stands (*SSCI*_obs_) and the SSCI predicted based on the structural complexity measured in the monoculture plots of the constituent species (*SSCI*_pred_). A positive *∆SSCI* value indicates that the structural complexity of a stand is greater than expected based on the complexity measured in the monoculture plots of its component species, while a negative value indicates the opposite. All tree mixtures had a greater productivity than expected based on monoculture yields (i.e., overyielding), and 87% of them were structurally more complex than would be expected on the basis of the weighted average complexity measured in monoculture plots (i.e., positive values of Δ*SSCI*; Fig. 4).

**Fig. 5.**
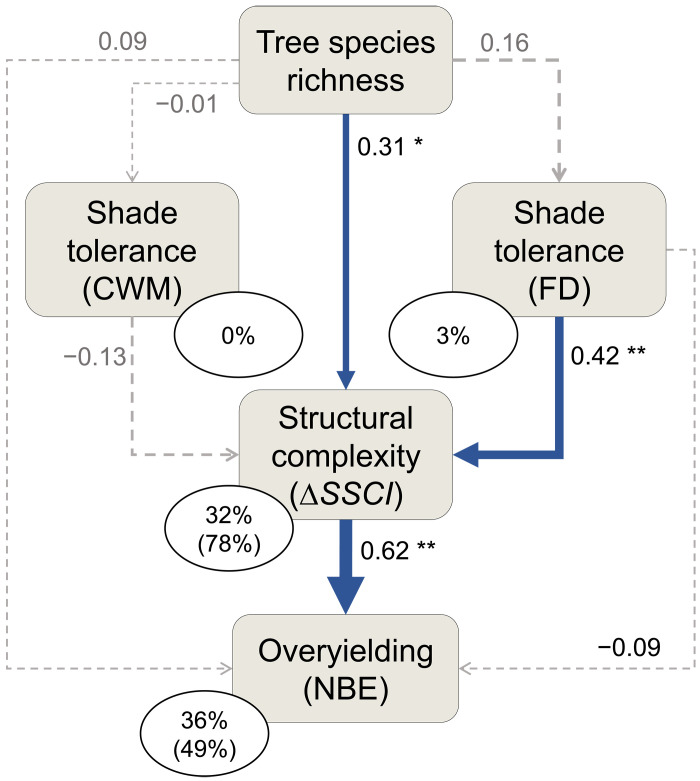
Structural equation model testing the role of shade tolerance in mediating biodiversity-complexity-productivity relationships in young tree communities. For each mixed-species community, we calculated the CWM and FD values of shade tolerance (ST). Δ*SSCI* represents the net effect of biodiversity on stand structural complexity (see Methods). The NBE on wood productivity was calculated following (*8*). The blue and red arrows indicate significant (*P < 0.05, **P < 0.01, and ***P < 0.001) positive and negative relationships, respectively. Arrow width is proportional to standardized path coefficients. Dashed gray arrows indicate nonsignificant (*P* > 0.10) paths. Numbers next to arrows are standardized path coefficients. Percentage values are proportions of variance explained by fixed effects; proportions of variance explained by fixed and random effects are in parentheses.

To understand the processes driving positive biodiversity-complexity-productivity relationships in our experiment, we fitted an additional SEM to our data. This SEM investigated the relative importance of shade tolerance (CWM and FD of this functional trait) in modulating the relationship between tree species richness and Δ*SSCI* (Fig. 5 and fig. S4). For each species included in the MyDiv experiment, shade tolerance index values were taken from (*38*). The SEM fitted our data well (Fisher’s *C* = 3.08, df = 4, *P* = 0.545) and explained 36% of the variation in overyielding. We found that the net positive effect of tree species richness on structural complexity (Δ*SSCI*) was largely attributable to variation in taxonomic diversity and shade tolerance within communities, which, in turn, led to greater overyielding in mixtures (Fig. 5). This positive relationship between community overyielding and Δ*SSCI* was mostly driven by greater complementarity effects in structurally more complex tree communities (Fig. 4), suggesting an important role for species interactions in mediating complexity-productivity relationships in forests. In line with this, our results showed that interspecific variation in shade tolerance (FD) was a stronger determinant of Δ*SSCI* (*P* = 0.008, *R*^2^ = 19%) than tree species richness (*P* = 0.012, *R*^2^ = 11%; Fig. 5). This indicates that species-rich communities composed of tree species with high and low shade tolerance were those associated with the strongest effects of biodiversity on structural complexity (fig. S4), which then led to stronger biodiversity effects on wood production (Fig. 5). The CWM of shade tolerance had no significant direct effect on Δ*SSCI* (CWM: *P* = 0.270). Tree species richness had no significant effect on the functional characteristics of communities, as neither the CWM nor FD values of shade tolerance were affected by tree species richness in our SEM (Fig. 5).

## DISCUSSION

The MyDiv tree biodiversity experiment allowed us to quantify the relative importance of aboveground and belowground processes in driving BPRs in young temperate forests. Our results show that stand structural complexity is a fundamental driver of positive BPRs in forests with different mycorrhizal associations. The positive and strong biodiversity-complexity-productivity relationship in mixed-species tree communities was primarily due to increased taxonomic diversity and complementary light-capture strategies among shade-tolerant and shade-intolerant species.

As hypothesized, stand structural complexity and AWP increased with tree species richness. However, we found no evidence to support our hypothesis that the strength of BPRs depends on mycorrhizal associations, indicating that mixing trees with different types of mycorrhizae does not necessarily lead to a greater increase in wood productivity in diverse communities, at least in the first 6 years after establishment. Previous studies in mature forests and long-term tree diversity experiments have shown that the positive effects of tree species richness on forest productivity depend on the proportion of different mycorrhizal associations within a community (*35*, *36*). However, the young developmental stage of our tree communities, nutrient legacies from previous agricultural use, and temporal variation in the importance of AM and EM trees in contributing to productivity in young mixed species communities (*33*) may explain why the effects of tree diversity on structural complexity and productivity were consistent across ecological strategies in our study.

We hypothesized that the positive effect of tree species richness on community productivity was mediated by (i) lower tree mortality and (ii) greater stand structural complexity. Although we found that plots with lower mortality rates were, on average, more productive, we found no relationship between tree species richness and tree mortality, suggesting that the expected reduction in tree mortality as a result of reduced intraspecific competition in mixtures did not occur during the first 6 years of our experiment (*39*). Given that plot-level mortality was comparatively low on average (median: 3.9%; mean: 7.7%), this may explain why we could not observe any significant mitigating effects of tree species richness on tree mortality. Instead, our results strongly support the hypothesis that community productivity is related to diversity-mediated shifts in stand structural complexity. We showed that the positive effects of tree diversity on wood productivity were strongly mediated by structural complexity within a stand: The higher the number of tree species in a plot, the greater the structural complexity and wood productivity of a forest stand. Although previous studies from tropical (*18*), subtropical (*16*), and temperate forests (*17*) using SSCI values derived from TLS data have also demonstrated that tree species richness promotes structurally complex stands, our findings provide evidence that variation in stand structural complexity acts as a central mechanism shaping BPRs in young tree communities. To further our understanding of the ecological mechanisms behind the positive relationships among biodiversity, structural complexity, and productivity in forests, the development of well-replicated experiments that explicitly manipulate tree species richness and stand structural complexity in a factorial design and in different forest types would be an important next step.

Our results also highlighted that the strength of the positive relationship between stand structural complexity and wood productivity depended on the light intensity measured at ground level. In particular, we found that the relationship between structural complexity and productivity was strongest in situations where light intensity at the forest floor was low, which suggests an important role for light interception in modulating structural complexity effects in forests. Our results strongly suggest that greater light interception by stand structural elements, particularly by foliage, is a necessary condition for structural complexity to increase wood productivity in young tree communities. This is also supported by the fact that we only observed a weak positive relationship between stand structural complexity and ground light intensity (fig. S5), indicating that processes that increase stand structural complexity and light interception are the most likely drivers of BPRs in our study. For example, greater structural complexity and light interception in mixtures may result from interspecific differences in tree growth and changes in outer and inner crown morphology and architecture to maximize light capture, leading to greater crown complementarity and greater wood volume growth (*5*, *20*). Although niche partitioning in mixtures generally reduces the intensity of competition among individuals, we did not observe any significant reduction in tree mortality rates in forest stands with a greater structural complexity.

Our finding that wood volume overyielding (i.e., NBE on AWP) was strongly controlled by stand structural complexity can be largely attributed to NBEs on structural complexity (Δ*SSCI*). In our experiment, 52 of 60 mixture plots were structurally more complex than expected based on the weighted average complexity of the monoculture plots of their constituent species. We showed that tree species richness and interspecific differences in shade tolerance did not directly increase overyielding but indirectly via enhancing the structural complexity of mixed-species communities. Given that a high CWM value of shade tolerance (i.e., communities dominated by shade tolerant species) did not translate into stronger biodiversity effects on complexity, directional shifts in the functional composition of tree communities did not explain the observed increase in stand structural complexity with increasing tree species richness. This contradicts predictions from the mass ratio theory (*40*) but suggests that mixing functionally different species is critical for shaping structural complexity in mixtures. In support of this, our results also highlight that mixing a greater number of tree species with different shade tolerance (i.e., increasing the taxonomic and functional diversity of a stand) enhances the net effect of biodiversity on complexity, with interspecific differences in shade tolerance being the most important in driving this effect. This is in agreement with several studies that have reported that dissimilarity in shade tolerance can increase forest productivity (*41*–*44*), suggesting that positive BPRs can result from greater complementarity in light-use strategies (*45*). This is consistent with our finding that the relationship between structural complexity and community productivity was strongest when light interception was high.

Shade tolerance is a key ecological trait that shapes plant-plant interactions because it is closely associated with functional traits related to a tree’s competitive ability for light (*46*, *47*). For instance, fast-growing species are often shade intolerant and vice versa (*48*), allowing shade-intolerant species to compensate for their lower ability to sustain competition by reaching the upper canopy layers more quickly (*49*). A higher variation in shade tolerance among species within a forest stand likely leads to more vertically structured canopies (*41*), where shade-intolerant species dominate the upper canopy layers, while shade-tolerant species populate lower canopy layers (*50*). This is in agreement with our finding that, among the tree species included in our experiment, those with greater shade tolerance were, on average, shorter than shade-intolerant species (fig. S6). This relationship was primarily driven by two ectomycorrhizal species with contrasting life history strategies: *Betula pendula*, fast growing and shade intolerant, and *Fagus sylvatica*, slow growing and shade tolerant [(*33*); fig. S6]. These differences between *B. pendula* and *F. sylvatica* caused greater vertical stratification in stands where both species were present, which led to stronger biodiversity effects on structural complexity (fig. S4) and wood productivity.

Our results showed that overyielding in mixed-species communities is primarily driven by stronger complementarity effects in structurally more complex tree communities, with selection effects playing a minor role. Greater complementarity effects in structurally more complex forest stands could be the result of more effective partitioning of light resources in patches with higher tree diversity. As a result, tree species growing in species-rich communities can occupy different sections along a light availability gradient, thereby increasing light interception and light-use efficiency of tree communities, which in turn increases productivity (*22*). Our results suggest that complexity-dependent interactions between tree species for light exploitation play an important role for complementarity effects to arise. In addition to species dissimilarity in shade tolerance, other components of functional diversity related to light capture, such as interspecific differences in tree branching and architecture, crown traits, or leaf phenology, may also have contributed to the net effect of biodiversity on stand structural complexity (*15*, *20*, *51*), which could also explain the importance of taxonomic diversity in mediating structural complexity in the forest plots of the MyDiv experiment.

Our results highlight the importance of selecting species with contrasting functional traits associated with light capture rather than contrasting mycorrhizal types in forest restoration projects to increase biomass accumulation during early stand development. Structurally complex stands can also support greater biodiversity, as the amount of niche opportunities for other trophic levels increases with heterogeneity in the horizontal and vertical structure of the forest (*52*, *53*). Therefore, improving stand structural complexity would benefit both biodiversity conservation and climate change mitigation, which is particularly relevant in the context of the UN Decade of (Forest) Restoration.

## MATERIALS AND METHODS

### Study site and experimental design

The study was conducted within the MyDiv biodiversity-ecosystem functioning experiment (*37*). The experimental site is located in Bad Lauchstädt (51°230N, 11°530E, Germany) at 114 to 116 m a.s.l with a continental summer-dry climate. The mean annual temperature and the mean annual precipitation are 8.8°C and 484 mm, respectively. Soils are developed on a parent material of silt over calcareous silt (loess), and the soil type is a Haplic Chernozem with a thick humus horizon. The study site is a former agricultural land. It was used as arable land for centuries until 2012 and then converted to grassland from 2013 to 2015. Because of these agricultural legacies and pedogenesis, the soil at our experimental site has a high nutrient content, particularly in nitrogen (*37*).

The MyDiv experiment was set up using a species pool of 10 native deciduous temperate tree species. These species were selected to represent two main types of mycorrhizal associations, with five tree species preferring AM and five tree species preferring EM [table S2 and (*31*)]. Two main factors have been factorially manipulated in this experiment: tree species richness (three levels: 1, 2, or 4 species per plot) and mycorrhizal associations (three levels: only AM tree species, only EM tree species, or mixture of AM and EM tree species). The experiment consists of 80 plots of 121 m^2^ (11 m by 11 m) arranged in two blocks. In March 2015, each plot was planted with 140 trees using a regular planting distance of 1 m. At the time of planting, all tree individuals were 2 to 3 years old. The tree diversity gradient included monocultures (*n* = 20), two-species mixtures (*n* = 30), and four-species mixtures (*n* = 30). For each species, monocultures were replicated twice. The species composition of two-species mixtures was not replicated (i.e., all two-species mixture plots had a unique species composition). In four-species mixtures with only one mycorrhizal type (AM or EM), all possible species combinations have been implemented twice. In four-species mixtures with both mycorrhizal types (AM + EM), however, only 10 unique species combinations were implemented. This experimental design resulted in 30 plots with AM tree species (*n* = 10 for each diversity level), 30 plots with EM tree species (*n* = 10 for each diversity level), and 20 plots with both mycorrhizal types (each *n* = 10 for two- and four-species mixtures). To ensure that all species were equally represented within a plot, individuals were planted in the same proportions and at the same overall density, with 70 (two-species mixtures) and 35 (four-species mixtures) individuals per species per plot. More details about the experimental design can be found in (*37*).

### Terrestrial laser scanning

TLS data were collected with a Riegel VZ400i terrestrial laser scanner (Riegl, Horn, Austria) in September 2021 under leaf-on conditions.

The scanner was set up on a tripod at a height of 1.3 m and positioned at the center of each plot. Two scans were taken per position to get a full spherical view. After taking the first vertical scan, the scanner was tilted by 90° and the horizontal scan was performed. The angular resolution of the scanner was set to 0.04, which corresponds to a resolution of 7 mm at a distance of 10 m with a laser frequency of 600 kHz. We chose these settings following (*54*) to have a better canopy penetration. All scans were taken under a clear sky in almost windless conditions.

The point clouds of the two scans of each center position were registered using RiSCAN PRO 10.11.3 (www.riegl.com) and then clipped to one specific plot to remove the information from the other plots. Filtering was conducted before registration, and stray and noise points were removed on the basis of pulse shape deviation and relative reflectance. In our study, all points with pulse shape deviation above 15 or reflectance less than 15 dB were removed following the manufacturer’s instructions to improve the quality of the point clouds (*55*). Because many trees already branched near the soil surface, we chose not to remove all points below 1.3 m [as done by Ehbrecht *et al.* (*12*)] but to remove only the soil surface layer of each plot.

### Quantifying the structural complexity of tree communities

For each plot, an index of stand structural complexity (*SSCI*) was calculated on the basis of a point cloud using the approach Ehbrecht *et al.* (*12*). This approach has been found to provide an effective measure to quantify the structural heterogeneity and complexity of a forest stand (*13*, *56*). The point cloud was converted to a voxel grid, and then the ratio of the total number of filled voxels to the total number of voxels was quantified (*57*). We used a voxel size of 5 cm and a slice thickness of 25 cm following (*16*). SSCI consists of two components (Eq. 1 and fig. S7): the effective number of layers (*ENL*) and the mean value of the fractal dimension of several cross sections of the point cloud (*MeanFrac*)

$$\mathrm{SSCI}={MeanFrac}^{\ln(ENL)}$$ (1)

*MeanFrac* is a fractal dimension index of the cross-sectional polygon, derived from a three-dimensional point cloud. It is dependent on the density of structural elements such as tree branches (*12*, *13*). *MeanFrac* was calculated as the mean value of the fractal dimension of 4500 cross sections. All points of each cross section were combined in a polygon after sorting by angle. *ENL* quantifies the effective number of layers within a cross section. It is closely related to the height of a tree and increases with increasing stand height (*16*, *56*). Thus, it captures the vertical stratification within a community. *ENL* was calculated using the inverse Simpson index. We computed *MeanFrac*, *ENL*, and SSCI using R 4.2.1 (*58*) with the packages VoxR (*59*) and sp (*60*).

### Quantifying the light interception of tree communities

In the year following TLS data acquisition (between 1 and 31 August 2022), light intensity was measured on an hourly basis in 40 study plots along the diversity gradient. Considering that the positive relationship between tree species richness and SSCI is known to strengthen over time (*16*), we do not believe that the fact that the TLS and light data were not taken in the same year calls into question the conclusions of our study. The light measurements were taken at a height of 20 cm using the HOBO Pendant Light 64 K Data Logger (ONSET, USA). Within each area, a square layout was set, with four sensors placed to capture variations in light distribution. To determine the average light intensity within each plot, the measurements from the sensors were averaged across August, excluding nighttime readings (i.e., considering only values above zero). This parameter was then used as a proxy for light interception by stand structural elements as lower ground light intensity values are expected in stands with high-light interception by structural elements such as leaves, branches, and stems (*61*, *62*). We only found a weak positive relationship between stand structural complexity and average light intensity at ground level (Pearson correlation coefficient: *r* = 0.29), which suggests that high-light interception of stand structural elements can be found in tree communities with low or high structural complexity. This coincides with findings that total light interception did not significantly vary in complex and less complex canopies (*62*, *63*).

### Tree growth data, mortality rate, and biodiversity effects

To avoid edge effects, growth analyses were focused on the 64 trees growing in the center (8 m by 8 m) of each of the 80 study plots. For each tree *i*, we measured stem diameter (*D_i_*, measured in meters at 5 cm aboveground) and tree height (*H_i_*, measured in meters as the distance between the stem base and the apical meristem). For each tree, wood volume (*V_i_*, m^3^) was then estimated as

$$V_{i}=(\pi D_{i}^{2})H_{i}f$$ (2)

where *f* is a cylindrical form factor of 0.5 (an average value for young broadleaved trees) to account for the deviation of the tree volume from the theoretical volume of a cylinder. For each plot *j*, AWP (m^3^ year^−1^) was calculated as the sum of the annual growth rates of all living trees (*n*) within a plot (Eq. 3). In Eq. 3, *V_ij,_*_1_ and *V_ij,_*_2_ are the wood volumes of tree *i* in plot *j* at the beginning (*t*_1_) and at the end (*t*_2_) of the growing period (2015–2021)

$${\mathrm{AWP}}_{j}=\sum_{i=1}^{n}\frac{V_{ij,2}-V_{ij,1}}{t_{2}-t_{1}}$$ (3)

Plot-level mortality was quantified as the relative mortality of all trees within a plot between 2015 and 2021. The net effect of biodiversity on AWP (NBE, also referred to as overyielding), as well as complementarity and selection effects, was calculated using the additive partitioning method of (*8*) implemented in the bef R package (*64*) available from GitHub (https://github.com/BenjaminDelory/bef).

### Quantifying the net effect of tree species diversity on stand structural complexity

The net effect of tree species diversity on stand structural complexity in plot *j* (∆*SSCI_j_*) was calculated using Eq. 4, where *SSCI_j,_*_obs_ is the SSCI measured in plot *j* by TLS, *SSCI_j,_*_pred_ is the SSCI value of plot *j* predicted based on the structural complexity measured in the monoculture plots of the species present in plot *j*, *S* is the tree species richness in plot *j*, *p_ij_* is the relative abundance of species *i* in plot *j* in 2021 (taking into account tree mortality from 2015 to 2021), and ${\bar{SSCI}}_{i,\mathrm{mono}}$ is the average SSCI measured in monocultures of species *i*. Positive *∆SSCI* values indicate that the structural complexity of a stand is greater than expected based on the complexity measured in the monoculture plots of its component species, while negative values indicate the opposite. Typically, positive *∆SSCI* values are expected if tree species richness has a positive effect on stand structural complexity

$$\text{Δ}{SSCI}_{j}={SSCI}_{j,\mathrm{obs}}-{SSCI}_{j,\mathrm{pred}}={SSCI}_{j,\mathrm{obs}}-\sum_{i=1}^{S}p_{ij}{\bar{SSCI}}_{i,\mathrm{mono}}$$ (4)

### Functional characteristics of tree communities

To understand better how tree species richness affects stand structural complexity (as measured by SSCI), we gathered data on shade tolerance for each tree species in our experiment. Their capacity to tolerate shade (as measured by a shade tolerance index) is a key trait related to species’ resource use strategy and ability to tolerate competition (*47*, *65*). Shade tolerance indices were taken from (*38*), and for each tree community, we calculated the community-weighted mean (CWM) and functional dispersion (FD) for shade tolerance. These metrics were calculated using the FD R package (*66*). Planting densities corrected for tree mortality between 2015 and 2021 were used to compute CWM and FD values.

### Statistical analysis

Before model fitting, tree species richness was log_2_-transformed, and communities’ tree mortality rates (%) measured between 2015 and 2021 were square root–transformed to meet model assumptions, which were visually checked and confirmed according to (*67*). We used linear mixed-effect models to assess the impact of tree species richness (numeric variable), mycorrhizal associations (categorical, three levels: AM, EM, and AM + EM), and their interacting effect on stand structural complexity (SSCI, continuous variable). Tree species composition of the plots was used as a random effect to avoid confounding effects between tree species richness and compositional differences in tree species among communities. Preliminary analyses indicated that there was no significant difference in SSCI between the two experimental blocks (type III sum of squares: *F* = 2.66, *P* = 0.117). Therefore, we did not use the study block as an additional covariate in the models. We used Hedges’ *g* effect size (*68*) as a standardized measure to quantify the strength of biodiversity effects (i.e., tree species richness) on SSCI. Effect sizes were calculated on the basis of predicted values of the best-fitted model at each level of tree species richness (monocultures, 2-species mixtures, and four-species mixtures) using pooled SD. Positive values of Hedges’ *g* indicate positive biodiversity effects on SSCI and vice versa. Small, moderate, and large effects are indicated by Hedges’ *g* values of 0.2, 0.5, and 0.8 (*69*).

To test the hypothesis that BPRs are modulated by mycorrhizal associations, we modeled AWP as a function of tree species richness and mycorrhizal associations, and their interaction as fixed effects. This linear mixed-effect model was fitted using tree species composition of the plots as a random effect. We found no evidence for a significant study block effect on AWP (type III sum of squares: *F* = 0.13, *P* = 0.717).

We modeled the relationship between SSCI and AWP using two linear mixed-effect models. In all models, tree species composition was used as a random effect. In the first model, AWP was modeled as a function of SSCI. In the second model, AWP was modeled as a function of SSCI (continuous fixed effect), average light intensity at ground level (continuous fixed effect), and their interaction. Adding tree species richness to the second model did not improve the model fit. All linear mixed-effect models were fitted with restricted maximum likelihood estimation.

To understand how tree species richness and mycorrhizal associations affected AWP in our young temperate forest plots, we fitted a SEM to our data using the piecewiseSEM R package (*70*). Our model includes four main pathways susceptible to modulate AWP: (i) a direct effect of tree species richness on AWP, (ii) an indirect effect of tree species richness on AWP via its effect on SSCI, (iii) an indirect effect of tree species richness on AWP via its effect on tree mortality, and (iv) a direct effect of the relative proportion of AM and EM trees in the community on AWP. This piecewise SEM consisted of a set of three mixed-effect models. All models were fitted using tree species composition as a random effect. The goodness of fit of our SEM was assessed using Fisher’s *C* test statistic (*70*).

To identify the most important drivers of *∆SSCI* (i.e., the net effect of tree species diversity on stand structural complexity) and its effect on overyielding, we fitted an additional piecewise SEM using the CWM and the FD of shade tolerance of the component species within a community. In each SEM, we tested three main pathways via which tree species richness could modulate *∆SSCI*: (i) a direct effect of tree species richness on *∆SSCI*, (ii) an indirect effect of tree species richness on *∆SSCI* via its effect on CWM, and (iii) an indirect effect of tree species richness on *∆SSCI* via its effect on FD. The direct effect of *∆SSCI* on NBE was represented in the model and a direct effect of tree species richness on NBE and a direct effect of FD on NBE. The SEM consisted of a set of two simple linear models and two mixed-effect models (tree species composition used as a random effect). The goodness of fit was assessed using Fisher’s *C* test statistic (*70*).

All statistical analyses were performed in R 4.2.1 (*58*) using the packages dplyr (*71*), emmeans (*72*), ggplot2 (*73*), ggeffects (*74*), lme4 (*75*), lmerTest (*76*), MuMIn (*77*), nlme (*78*), variancePartition (*79*), VoxR (*59*), and sp (*60*).

## Supplementary Materials

### This PDF file includes:

Tables S1 and S2 Figs. S1 to S7 References
