# Supplementary material for: Tree diversity increases productivity through enhancing structural complexity across mycorrhizal types
Source: Sci Adv. 2023 Oct 6;9(40):eadi2362. doi: 10.1126/sciadv.adi2362 (PMC10558120; doi:10.1126/sciadv.adi2362)
Supplement: Supplementary file 1 — Tables S1 and S2 Figs. S1 to S7 References [file sciadv.adi2362_sm.pdf]

Science Advances
AAAS

Supplementary Materials for

Tree diversity increases productivity through enhancing structural complexity across mycorrhizal types

Tama Ray *et al.*

Corresponding author: Tama Ray, tama.ray@mailbox.tu-dresden.de; Andreas Fichtner, andreas.fichtner@leuphana.de; goddert_v_oheimb@tu-dresden.de

Sci. Adv. **9**, eadi2362 (2023)
DOI: 10.1126/sciadv.adi2362

This PDF file includes:

Tables S1 to S2
Figs. S1 to S7
Reference

Table S1. Results of linear mixed-effects models of the effects of tree species richness on stand structural complexity (SSCI) and community productivity (AWP). SD, standard deviation. The variance explained by the fixed effects alone (marginal R^2) and by both the fixed and random effects (conditional R^2) was calculated according to (*80*). See Table 1 for further information.

	SSCI-model	AWP-model	AWP-model (subsetted data)
**Random effects**			
SD (species composition)	0.970	0.023	0.023
SD (residuals)	0.340	0.022	0.012
**Model fit**			
R^2_m	0.19	0.11	0.48
R^2_c	0.91	0.56	0.89

Table S2. List of tree species used in the MyDiv experiment and their associated functional characteristics, including mycorrhizal associations, net primary productivity in monoculture and shade tolerance indices. Within each category of tree species' preferred mycorrhizal association, species are ranked from most to least productive. AM, arbuscular mycorrhiza; EM, ectomycorrhiza; AWP, annual wood productivity.

Tree species	Mycorrhizal association	AWP (m^3/year) in monoculture	Shade tolerance index
Prunus avium	AM	0.15	3.33
Acer pseudoplatanus	AM	0.14	3.73
Fraxinus excelsior	AM	0.10	2.66
Sorbus aucuparia	AM	0.08	2.73
Aesculus hippocastanum	AM	0.07	3.43
Tilia platyphyllos	EM	0.16	4.00
Betula pendula	EM	0.11	2.03
Carpinus betulus	EM	0.10	3.97
Quercus petraea	EM	0.04	2.73
Fagus sylvatica	EM	0.04	4.56

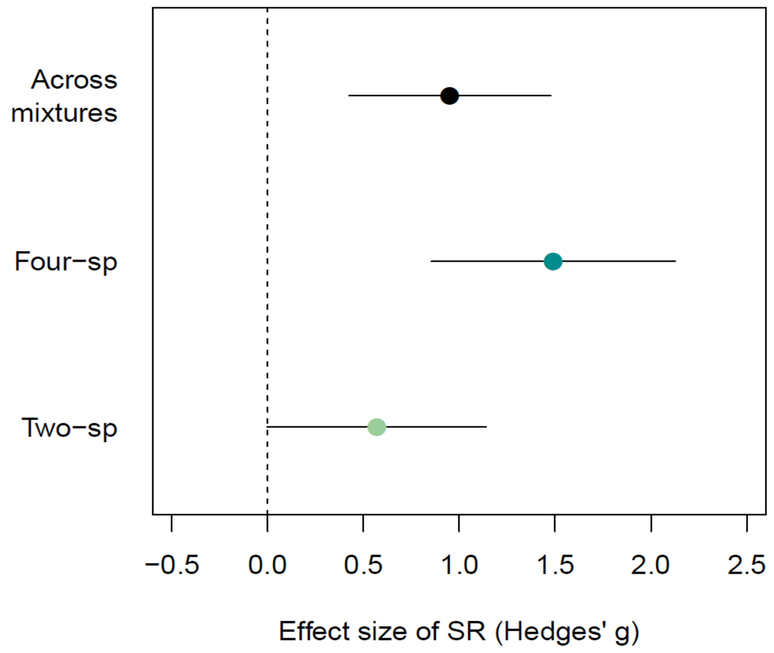

Fig. S1. **Effect sizes for the biodiversity-complexity relationship showing how tree species richness affects the strength of biodiversity effects (Hedges' g effect size) on stand structural complexity (SSCI).** Points are predicted means of mixed-effects models, and error bars denote the 95% confidence intervals. Positive values indicate a higher structural complexity in mixed-species communities compared to monocultures, while negative values indicate the opposite. Error bars not overlapping with zero indicate significant biodiversity effects, and vice versa. Across mixtures: 2- and 4-species mixtures.

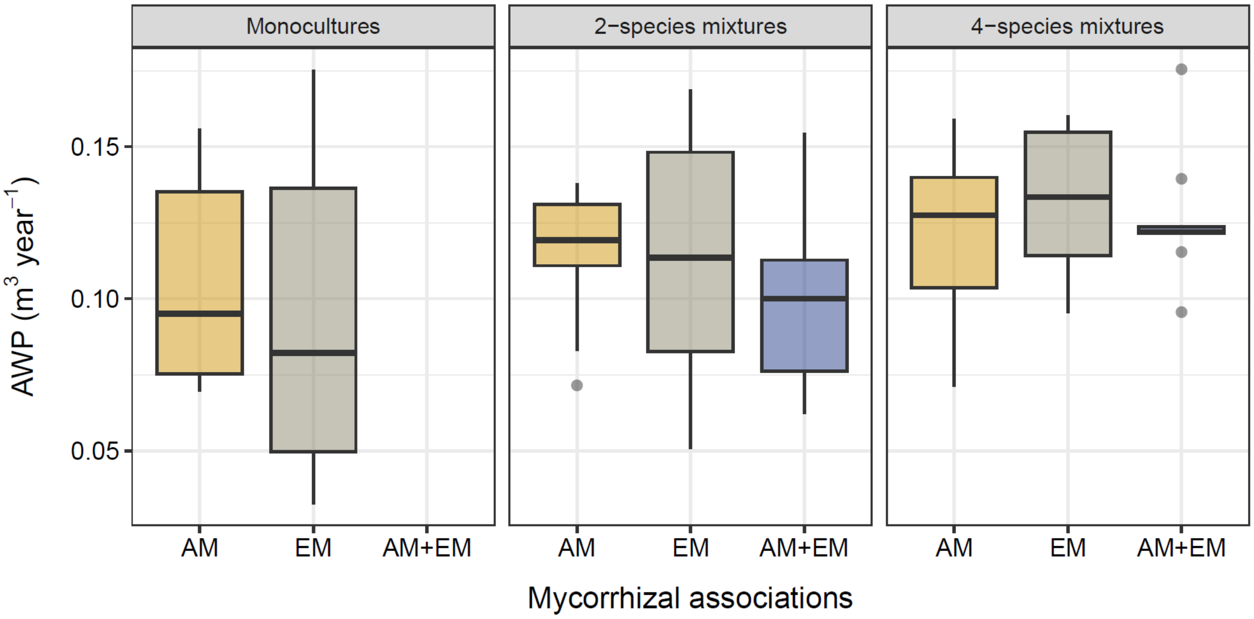

Fig. S2. **Effects of mycorrhizal associations on community productivity does not depend on tree species richness.** Boxplots show the median (horizontal white lines), the 25% and 75% percentiles (edges of the box) and 1.5 times the interquartile range (whiskers) of observed community productivity. Open circles indicate productivity values that are greater or smaller than 1.5 times the interquartile range. Differences among mycorrhizal associations were not statistically significant (Tukey-Test: $P > 0.10$). Note that the interaction between tree species richness and mycorrhizal associations was not significant ($P = 0.54$). AM: arbuscular mycorrhizal tree species; EM: ectomycorrhizal tree species.

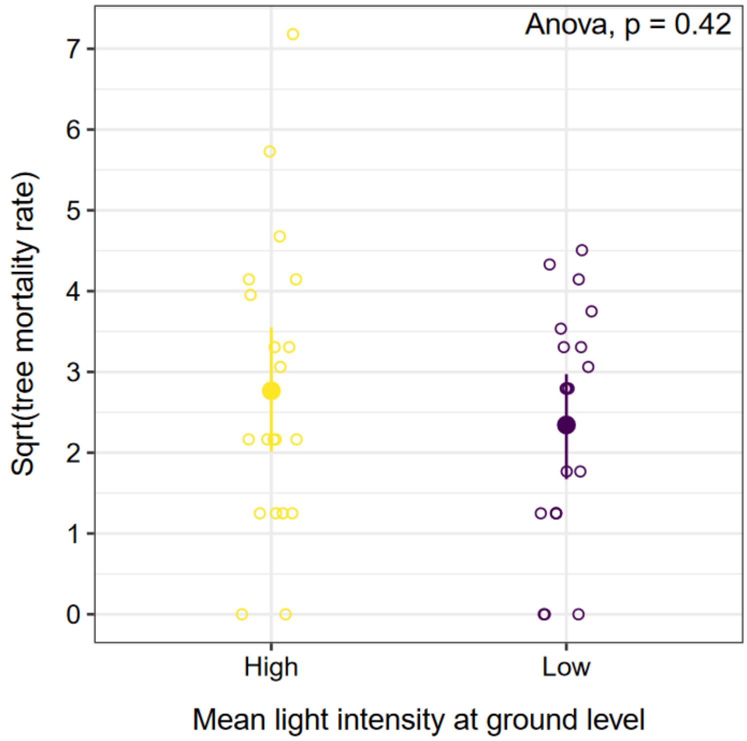

Fig. S3. Mortality rate does not significantly vary with different levels of light intensities. Low and high light intensity at ground level was computed as the 25% and 75% quantile of measured light intensity. The points correspond to observed values of tree mortality rate (%) within a stand.

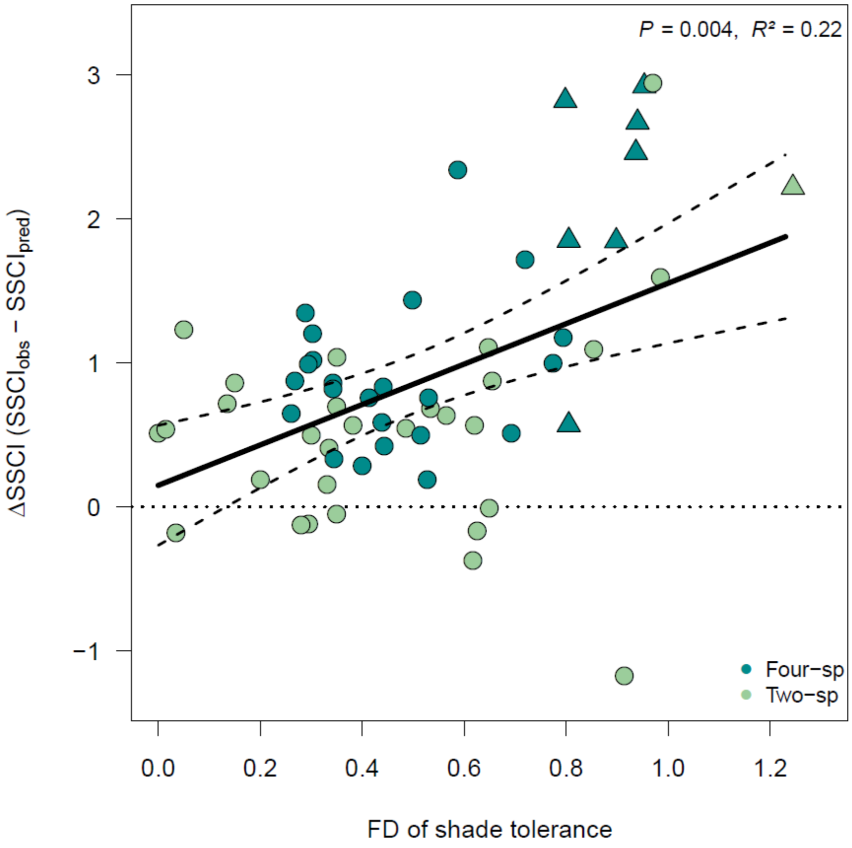

Fig. S4. Shade tolerance-structural complexity relationship. Relationship between the net effect of tree species richness on structural complexity (ΔSSCI) and the functional dispersion (FD) of shade tolerance within mixtures. For each mixture, ΔSSCI was calculated as the difference between observed ($SSCI_{obs}$) and predicted ($SSCI_{pred}$) structural complexity (see Methods). The solid line is a mixed-effect model fit. The dotted lines indicate the 95% confidence interval of the prediction. Raw data are shown with individual data points, with triangles showing plots containing both *Betula pendula* and *Fagus sylvatica* growing in 2-species (light green) or 4-species (dark green) mixtures. The R^2-value refers to the proportion of variance explained by FD.

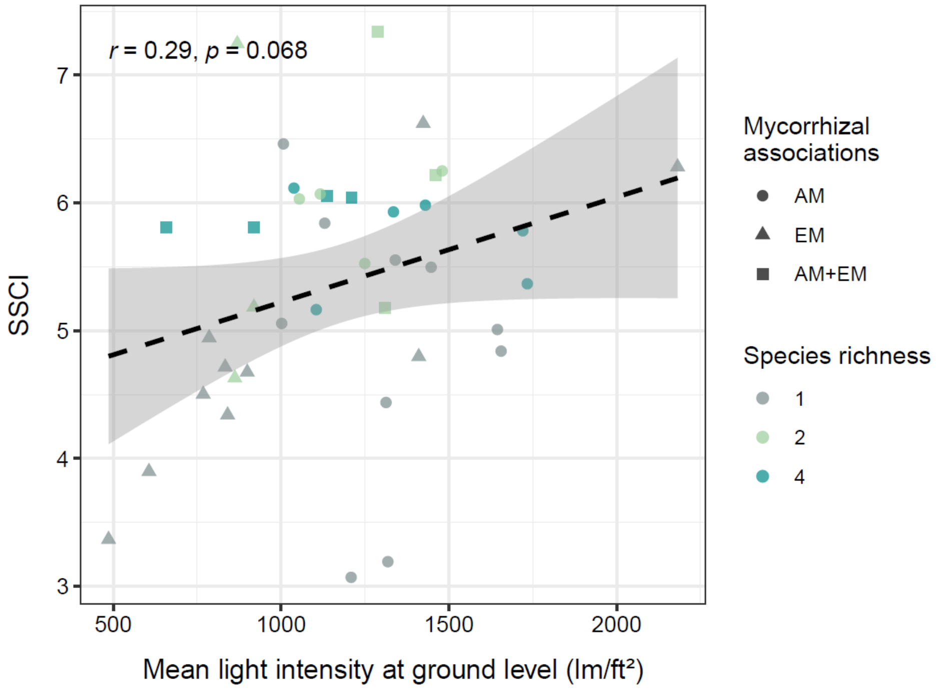

Fig. S5. Light condition-structural complexity relationship. Correlation between the mean light intensity at ground level (used as a proxy for stand-level light interception), and stand structural complexity (SSCI) for a subset of plots (*n*=40). The solid line is a linear model fit, and the shaded area indicates the 95% confidence interval of the prediction. Raw data are shown with individual data points. Different colors indicate tree species richness levels and different shapes correspond to mycorrhizal associations.

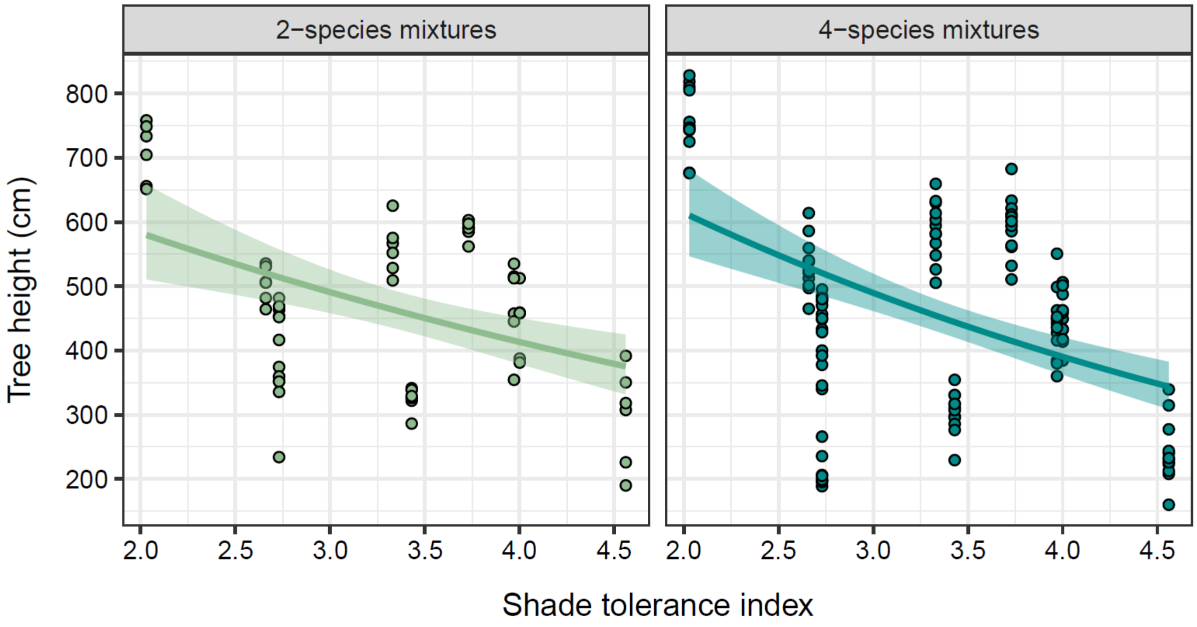

Fig. S6. Effects of shade tolerance on tree height growth in mixed-species tree communities. Univariate relationships are displayed for 2-species mixtures and 4-species mixtures. Individual dots represent the average height in 2021 (year of scanning) of a species in a plot. Lines are generalized linear model fits (Gamma distribution and log link function). Shade tolerance was used as a continuous variable in the model, while species richness was used as a factor with two levels. There was no interaction between tree species richness and shade tolerance (P = 0.377), but shade tolerance had a strong negative effect on tree height (P = 0.0008). Across all species included in our experiment, tree species richness did not affect tree height (P = 0.441).

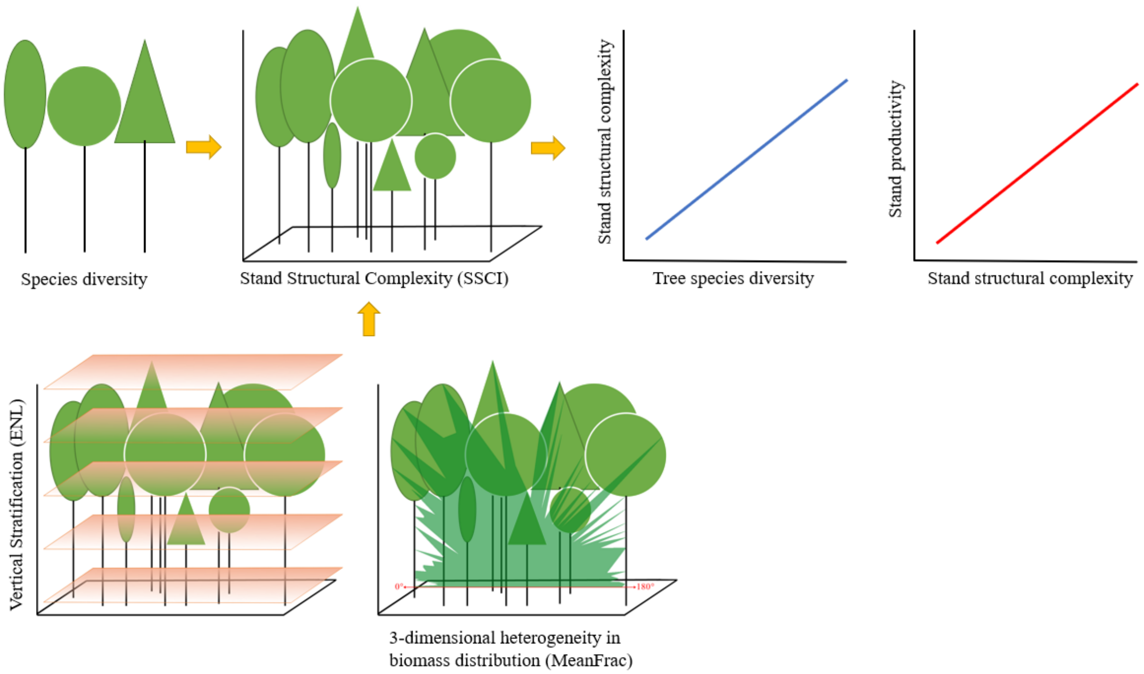

Fig. S7. Graphical representation of tree species diversity effects on stand productivity, enhanced by stand structural complexity. Greater tree species richness allows different species to grow in different strata depending on their degree of morphological, architectural and functional dissimilarity. This promotes greater vertical stratification (ENL) and 3-dimensional heterogeneity in biomass distribution (MeanFrac) of the stand, two basic components of stand structural complexity. Therefore, we hypothesized that the structural complexity of a tree community enhances resource acquisition of the trees and helps to store wood biomass in different strata of the stand.

REFERENCES AND NOTES

1. Y. Huang, Y. Chen, N. Castro-Izaguirre, M. Baruffol, M. Brezzi, A. Lang, Y. Li, W. Härdtle, G. von Oheimb, X. Yang, X. Liu, K. Pei, S. Both, B. Yang, D. Eichenberg, T. Assmann, J. Bauhus, T. Behrens, F. Buscot, X.-Y. Chen, D. Chesters, B.-Y. Ding, W. Durka, A. Erfmeier, J. Fang, M. Fischer, L.-D. Guo, D. Guo, J. L. M. Gutknecht, J.-S. He, C.-L. He, A. Hector, L. Hönig, R.-Y. Hu, A.-M. Klein, P. Kühn, Y. Liang, S. Li, S. Michalski, M. Scherer-Lorenzen, K. Schmidt, T. Scholten, A. Schuldt, X. Shi, M.-Z. Tan, Z. Tang, S. Trogisch, Z. Wang, E. Welk, C. Wirth, T. Wubet, W. Xiang, M. Yu, X.-D. Yu, J. Zhang, S. Zhang, N. Zhang, H.-Z. Zhou, C.-D. Zhu, L. Zhu, H. Bruelheide, K. Ma, P. A. Niklaus, B. Schmid, Impacts of species richness on productivity in a large-scale subtropical forest experiment. *Science* **362**, 80–83 (2018).

2. J. Liang, T. W. Crowther, N. Picard, S. Wiser, M. Zhou, G. Alberti, E.-D. Schulze, A. D. McGuire, F. Bozzato, H. Pretzsch, S. de-Miguel, A. Paquette, B. Hérault, M. Scherer-Lorenzen, C. B. Barrett, H. B. Glick, G. M. Hengeveld, G.-J. Nabuurs, S. Pfautsch, H. Viana, A. C. Vibrans, C. Ammer, P. Schall, D. Verbyla, N. Tchebakova, M. Fischer, J. V. Watson, H. Y. H. Chen, X. Lei, M.-J. Schelhaas, H. Lu, D. Gianelle, E. I. Parfenova, C. Salas, E. Lee, B. Lee, H. S. Kim, H. Bruelheide, D. A. Coomes, D. Piotto, T. Sunderland, B. Schmid, S. Gourlet-Fleury, B. Sonké, R. Tavani, J. Zhu, S. Brandl, J. Vayreda, F. Kitahara, E. B. Searle, V. J. Neldner, M. R. Ngugi, C. Baraloto, L. Frizzera, R. Bałazy, J. Oleksyn, T. Zawiła-Niedźwiecki, O. Bouriaud, F. Bussotti, L. Finér, B. Jaroszewicz, T. Jucker, F. Valladares, A. M. Jagodzinski, P. L. Peri, C. Gonmadje, W. Marthy, T. O’Brien, E. H. Martin, A. R. Marshall, F. Rovero, R. Bitariho, P. A. Niklaus, P. Alvarez-Loayza, N. Chamuya, R. Valencia, F. Mortier, V. Wortel, N. L. Engone-Obiang, L. V. Ferreira, D. E. Odeke, R. M. Vasquez, S. L. Lewis, P. B. Reich, Positive biodiversity-productivity relationship predominant in global forests. *Science* **354**, aaf8957 (2016).

3. A. Paquette, A. Hector, B. Castagneyrol, M. Vanhellemont, J. Koricheva, M. Scherer-Lorenzen, K. Verheyen; TreeDivNet, A million and more trees for science. *Nat. Ecol. Evol.* **2**, 763–766 (2018).

4. A. Fichtner, W. Härdtle, Y. Li, H. Bruelheide, M. Kunz, G. von Oheimb, From competition to facilitation: How tree species respond to neighbourhood diversity. *Ecol. Lett.* **20**, 892–900 (2017).

5. M. Kunz, A. Fichtner, W. Härdtle, P. Raumonen, H. Bruelheide, G. Oheimb, Neighbour species richness and local structural variability modulate aboveground allocation patterns and crown morphology of individual trees. *Ecol. Lett.* **22**, 2130–2140 (2019).

6. J. Guillemot, M. Kunz, F. Schnabel, A. Fichtner, C. P. Madsen, T. Gebauer, W. Härdtle, G. von Oheimb, C. Potvin, Neighbourhood-mediated shifts in tree biomass allocation drive overyielding in tropical species mixtures. *New Phytol.* **228**, 1256–1268 (2020).

7. G. Gheyret, H.-T. Zhang, Y. Guo, T.-Y. Liu, Y.-H. Bai, S. Li, B. Schmid, H. Bruelheide, K. Ma, Z. Tang, Radial growth response of trees to seasonal soil humidity in a subtropical forest. *BAsic Appl. Ecol.* **55**, 74–86 (2021).

8. M. Loreau, A. Hector, Partitioning selection and complementarity in biodiversity experiments. *Nature* **412**, 72–76 (2001).

9. C. McElhinny, P. Gibbons, C. Brack, An objective and quantitative methodology for constructing an index of stand structural complexity. *For. Ecol. Manage.* **235**, 54–71 (2006).

10. E. A. LaRue, J. A. Knott, G. M. Domke, H. Y. Chen, Q. Guo, M. Hisano, C. Oswalt, S. Oswalt, N. Kong, K. M. Potter, S. Fei, Structural diversity as a reliable and novel predictor for ecosystem productivity. *Front. Ecol. Environ.* **21**, 33–39 (2023).

11. E. R. Lines, F. J. Fischer, H. J. F. Owen, T. Jucker, The shape of trees: Reimagining forest ecology in three dimensions with remote sensing. *J. Ecol.* **110**, 1730–1745 (2022).

12. M. Ehbrecht, P. Schall, C. Ammer, D. Seidel, Quantifying stand structural complexity and its relationship with forest management, tree species diversity and microclimate. *Agric. For. Meteorol.* **242**, 1–9 (2017).

13. M. Ehbrecht, D. Seidel, P. Annighöfer, H. Kreft, M. Köhler, D. C. Zemp, K. Puettmann, R. Nilus, F. Babweteera, K. Willim, M. Stiers, D. Soto, H. J. Boehmer, N. Fisichelli, M. Burnett, G. Juday, S. L. Stephens, C. Ammer, Global patterns and climatic controls of forest structural complexity. *Nat. Commun.* **12**, 519 (2021).

14. T. C. Coverdale, A. B. Davies, Unravelling the relationship between plant diversity and vegetation structural complexity: A review and theoretical framework. *J. Ecol.* **111**, 1378–1395 (2023).

15. H. Pretzsch, Canopy space filling and tree crown morphology in mixed-species stands compared with monocultures. *For. Ecol. Manage.* **327**, 251–264 (2014).

16. M. D. Perles-Garcia, M. Kunz, A. Fichtner, W. Härdtle, G. von Oheimb, Tree species richness promotes an early increase of stand structural complexity in young subtropical plantations. *J. Appl. Ecol.* **58**, 2305–2314 (2021).

17. J. Juchheim, M. Ehbrecht, P. Schall, C. Ammer, D. Seidel, Effect of tree species mixing on stand structural complexity. *Forestry: An International Journal of Forest Research*, **cpz046** (2019).

18. D. C. Zemp, M. Ehbrecht, D. Seidel, C. Ammer, D. Craven, J. Erkelenz, B. Irawan, L. Sundawati, D. Hölscher, H. Kreft, Mixed-species tree plantings enhance structural complexity in oil palm plantations. *Agr Ecosyst Environ* **283**, 106564 (2019).

19. L. J. Williams, A. Paquette, J. Cavender-Bares, C. Messier, P. B. Reich, Spatial complementarity in tree crowns explains overyielding in species mixtures. *Nat. Ecol. Evol.* **1**, 0063 (2017).

20. M. Hildebrand, M. D. Perles-Garcia, M. Kunz, W. Härdtle, G. von Oheimb, A. Fichtner, Tree-tree interactions and crown complementarity: The role of functional diversity and branch traits for canopy packing. *Basic Appl. Ecol.* **50**, 217–227 (2021).

21. M. M. Duarte, R. De A. Moral, J. Guillemot, C. I. F. Zuim, C. Potvin, W. H. Bonat, J. L. Stape, P. H. S. Brancalion, High tree diversity enhances light interception in tropical forests. *J. Ecol.* **109**, 2597–2611 (2021).

22. L. J. Williams, E. E. Butler, J. Cavender-Bares, A. Stefanski, K. E. Rice, C. Messier, A. Paquette, P. B. Reich, Enhanced light interception and light use efficiency explain overyielding in young tree communities. *Ecol. Lett.* **24**, 996–1006 (2021).

23. A. Dănescu, A. T. Albrecht, J. Bauhus, Structural diversity promotes productivity of mixed, uneven-aged forests in southwestern Germany. *Oecologia* **182**, 319–333 (2016).

24. C. M. Gough, J. W. Atkins, R. T. Fahey, B. S. Hardiman, High rates of primary production in structurally complex forests. *Ecology* **100** e02864 (2019).

25. B. S. Hardiman, G. Bohrer, C. M. Gough, C. S. Vogel, P. S. Curtis, The role of canopy structural complexity in wood net primary production of a maturing northern deciduous forest. *Ecology* **92**, 1818–1827 (2011).

26. S. E. Smith, D. J. Read, *Mycorrhizal Symbiosis* (Academic Press, 2010).

27. G. T. Freschet, C. Roumet, L. H. Comas, M. Weemstra, A. G. Bengough, B. Rewald, R. D. Bardgett, G. B. De Deyn, D. Johnson, J. Klimešová, M. Lukac, M. L. McCormack, I. C. Meier, L. Pagès, H. Poorter, I. Prieto, N. Wurzburger, M. Zadworny, A. Bagniewska-Zadworna, E. B. Blancaflor, I. Brunner, A. Gessler, S. E. Hobbie, C. M. Iversen, L. Mommer, C. Picon-Cochard, J. A. Postma, L. Rose, P. Ryser, M. Scherer-Lorenzen, N. A. Soudzilovskaia, T. Sun, O. J. Valverde-Barrantes, A. Weigelt, L. M. York, A. Stokes, Root traits as drivers of plant and ecosystem functioning: Current understanding, pitfalls and future research needs. *New Phytol.* **232**, 1123–1158 (2021).

28. V. B. Chaudhary, E. P. Holland, S. Charman-Anderson, A. Guzman, L. Bell-Dereske, T. E. Cheeke, A. Corrales, J. Duchicela, C. Egan, M. M. Gupta, S. E. Hannula, R. Hestrin, S. Hoosein, A. Kumar, G. Mhretu, L. Neuenkamp, P. Soti, Y. Xie, T. Helgason, What are mycorrhizal traits? *Trends Ecol. Evol.* **37**, 573–581 (2022).

29. P. Bonfante, A. Genre, Mechanisms underlying beneficial plant–fungus interactions in mycorrhizal symbiosis. *Nat. Commun.* **1**, 48 (2010).

30. F. P. Teste, M. D. Jones, I. A. Dickie, Dual-mycorrhizal plants: Their ecology and relevance. *New Phytol.* **225**, 1835–1851 (2020).

31. H. Heklau, N. Schindler, F. Buscot, N. Eisenhauer, O. Ferlian, L. D. Prada Salcedo, H. Bruelheide, Mixing tree species associated with arbuscular or ectotrophic mycorrhizae reveals dual mycorrhization and interactive effects on the fungal partners. *Ecol. Evol.* **11**, 5424–5440 (2021).

32. B. D. Lindahl, A. Tunlid, Ectomycorrhizal fungi – potential organic matter decomposers, yet not saprotrophs. *New Phytol.* **205**, 1443–1447 (2015).

33. P. Dietrich, O. Ferlian, Y. Huang, S. Luo, J. Quosh, N. Eisenhauer, Tree diversity effects on productivity depend on mycorrhizae and life strategies in a temperate forest experiment. *Ecology* **104**, e3896 (2023).

34. Z. Mao, F. van der Plas, A. Corrales, K. J. Anderson-Teixeira, N. A. Bourg, C. Chu, Z. Hao, G. Jin, J. Lian, F. Lin, B. Li, W. Luo, W. J. McShea, J. A. Myers, G. Shen, X. Wang, E. Yan, J. Ye, W. Ye, Z. Yuan, X. Wang, Scale-dependent diversity–biomass relationships can be driven by tree mycorrhizal association and soil fertility. *Ecological Monographs* **93**, 10.1002/ecm.1568 (2023).

35. S. Luo, R. P. Phillips, I. Jo, S. Fei, J. Liang, B. Schmid, N. Eisenhauer, Higher productivity in forests with mixed mycorrhizal strategies. *Nat. Commun.* **14**, 1377 (2023).

36. M. Deng, S. Hu, L. Guo, L. Jiang, Y. Huang, B. Schmid, C. Liu, P. Chang, S. Li, X. Liu, K. Ma, L. Liu, Tree mycorrhizal association types control biodiversity-productivity relationship in a subtropical forest. *Sci. Adv.* **9**, , eadd4468 (2023).

37. O. Ferlian, S. Cesarz, D. Craven, J. Hines, K. E. Barry, H. Bruelheide, F. Buscot, S. Haider, H. Heklau, S. Herrmann, P. Kühn, U. Pruschitzki, M. Schädler, C. Wagg, A. Weigelt, T. Wubet, N. Eisenhauer, Mycorrhiza in tree diversity–ecosystem function relationships: Conceptual framework and experimental implementation. *Ecosphere* **9**, e02226 (2018).

38. Ü. Niinemets, F. Valladares, Tolerance to shade, drought, and waterlogging of temperate northern hemisphere trees and shrubs. *Ecol. Manogr.* **76**, 521–547 (2006).

39. J. S. Clark, Individuals and the variation needed for high species diversity in forest trees. *Science* **327**, 1129–1132 (2010).

40. J. P. Grime, Benefits of plant diversity to ecosystems: Immediate, filter and founder effects. *J. Ecol.* **86**, 902–910 (1998).

41. X. Morin, L. Fahse, M. Scherer-Lorenzen, H. Bugmann, Tree species richness promotes productivity in temperate forests through strong complementarity between Species. *Ecol. Lett.* **14**, 1211–1219 (2011).

42. Y. Zhang, H. Y. H. Chen, P. B. Reich, Forest productivity increases with evenness, species richness and trait variation: A global meta-analysis. *J. Ecol.* **100**, 742–749 (2012).

43. M. Toïgo, T. Perot, B. Courbaud, B. Castagneyrol, J.-C. Gégout, F. Longuetaud, H. Jactel, P. Vallet, Difference in shade tolerance drives the mixture effect on oak productivity. *J. Ecol.* **106**, 1073–1082 (2018).

44. E. B. Searle, H. Y. H. Chen, Complementarity effects are strengthened by competition intensity and global environmental change in the central boreal forests of Canada. *Ecol. Lett.* **23**, 79–87 (2020).

45. T. Jucker, O. Bouriaud, D. Avacaritei, I. Dănilă, G. Duduman, F. Valladares, D. A. Coomes, Competition for light and water play contrasting roles in driving diversity-productivity relationships in Iberian forests. *J. Ecol.* **102**, 1202–1213 (2014).

46. F. Valladares, Ü. Niinemets, Shade tolerance, a key plant feature of complex nature and consequences. *Annu. Rev. Ecol. Evol. Syst.* **39**, 237–257 (2008).

47. G. Kunstler, D. Falster, D. A. Coomes, F. Hui, R. M. Kooyman, D. C. Laughlin, L. Poorter, M. Vanderwel, G. Vieilledent, S. J. Wright, M. Aiba, C. Baraloto, J. Caspersen, J. H. C. Cornelissen, S. Gourlet-Fleury, M. Hanewinkel, B. Herault, J. Kattge, H. Kurokawa, Y. Onoda, J. Peñuelas, H. Poorter, M. Uriarte, S. Richardson, P. Ruiz-Benito, I.-F. Sun, G. Ståhl, N. G. Swenson, J. Thompson, B. Westerlund, C. Wirth, M. A. Zavala, H. Zeng, J. K. Zimmerman, N. E. Zimmermann, M. Westoby, Plant functional traits have globally consistent effects on competition. *Nature* **529**, 204–207 (2016).

48. K. M. Sendall, C. H. Lusk, P. B. Reich, Trade-offs in juvenile growth potential vs. shade tolerance among subtropical rain forest trees on soils of contrasting fertility. *Funct Ecol.* **30**, 845–855 (2016).

49. I. R. Gilbert, P. G. Jarvis, H. Smith, Proximity signal and shade avoidance differences between early and late successional trees. *Nature* **411**, 792–795 (2001).

50. Ü. Niinemets, A review of light interception in plant stands from leaf to canopy in different plant functional types and in species with varying shade tolerance. *Ecol. Res.* **25**, 693–714 (2010).

51. J. Sapijanskas, A. Paquette, C. Potvin, N. Kunert, M. Loreau, Tropical tree diversity enhances light capture through crown plasticity and spatial and temporal niche differences. *Ecology* **95**, 2479–2492 (2014).

52. A. Schuldt, A. Ebeling, M. Kunz, M. Staab, C. Guimarães-Steinicke, D. Bachmann, N. Buchmann, W. Durka, A. Fichtner, F. Fornoff, W. Härdtle, L. R. Hertzog, A.-M. Klein, C. Roscher, J. Schaller, G. von Oheimb, A. Weigelt, W. Weisser, C. Wirth, J. Zhang, H. Bruelheide, N. Eisenhauer, Multiple plant diversity components drive consumer communities across ecosystems. *Nat. Commun.* **10**, 1460 (2019).

53. L. Heidrich, S. Bae, S. Levick, S. Seibold, W. Weisser, P. Krzystek, P. Magdon, T. Nauss, P. Schall, A. Serebryanyk, S. Wöllauer, C. Ammer, C. Bässler, I. Doerfler, M. Fischer, M. M. Gossner, M. Heurich, T. Hothorn, K. Jung, H. Kreft, E.-D. Schulze, N. Simons, S. Thorn, J. Müller, Heterogeneity–diversity relationships differ between and within trophic levels in temperate forests. *Nat. Ecol. Evol.* **4**, 1204–1212 (2020).

54. K. F. Reich, M. Kunz, G. von Oheimb, A new index of forest structural heterogeneity using tree architectural attributes measured by terrestrial laser scanning. *Ecol. Indic.* **133**, 108412 (2021).

55. M. Pfennigbauer, A. Ullrich, Improving quality of laser scanning data acquisition through calibrated amplitude and pulse deviation measurement, in M. D. Turner, G. W. Kamerman, Eds. (Orlando, Florida, 2010);), p. 76841F.

56. K. Willim, C. Ammer, D. Seidel, P. Annighöfer, J. Schmucker, P. Schall, M. Ehbrecht, Short - term dynamics of structural complexity in differently managed and unmanaged European beech forests. *Trees, Forests and People* **8**, 100231 (2022).

57. M. Ehbrecht, P. Schall, J. Juchheim, C. Ammer, D. Seidel, Effective number of layers: A new measure for quantifying three-dimensional stand structure based on sampling with terrestrial LiDAR. *For. Ecol. Manage.* **380**, 212–223 (2016).

58. R Core Team, R: A language and environment for statistical computing (2022); www.R-project.org/.

59. B. Lecigne, S. Delagrange, C. Messier, Exploring trees in three dimensions: VoxR, a novel voxel-based R package dedicated to analysing the complex arrangement of tree crowns. *Ann. Bot.* **121**, 589–601 (2018).

60. R. Bivand, E. J. Pebesma, V. Gómez-Rubio, *Applied spatial data analysis with R* (Springer, ed. 2, 2013).

61. N. J. J. Bredé, Ground-based measurements of leaf area index: A review of methods, instruments and current controversies. *J. Exp. Bot.* **54**, 2403–2417 (2003).

62. K. Rissanen, M.-O. Martin-Guay, A.-S. Riopel-Bouvier, A. Paquette, Light interception in experimental forests affected by tree diversity and structural complexity of dominant canopy. *Agric. For. Meteorol.* **278**, 107655 (2019).

63. A. T. Fotis, P. S. Curtis, Effects of structural complexity on within-canopy light environments and leaf traits in a northern mixed deciduous forest, *Tree Physiol.*, **37**, 1426–1435 (2017).

64. B. M. Delory, E. W. A. Weidlich, P. von Gillhaussen, V. M. Temperton, When history matters: The overlooked role of priority effects in grassland overyielding. *Funct Ecol.* **33**, 2369–2380 (2019).

65. P. B. Reich, The world-wide 'fast-slow' plant economics spectrum: A traits manifesto. *J. Ecol.* **102**, 275–301 (2014).

66. E. Laliberté, P. Legendre, A distance-based framework for measuring functional diversity from multiple traits. *Ecology* **91**, 299–305 (2010).

67. A. F. Zuur, Ed., *Mixed effects models and extensions in ecology with R* (Springer, New York, 2009), 2009.

68. L. V. Hedges, I. Olkin, *Statistical Methods for Meta-Analysis* (Academic Press, 1985).

69. J. Koricheva, J. Gurevitch, K. Mengersen, *Handbook of Meta-analysis in Ecology and Evolution* (Princeton Univ. Press, 2013).

70. J. S. Lefcheck, piecewiseSEM: Piecewise structural equation modelling in R for ecology, evolution, and systematics. *Methods Ecol. Evol.* **7**, 573–579 (2016).

71. H. Wickham, R. François, L. Henry, K. Müller, dplyr: A grammar of data manipulation (2022); https://CRAN.R-project.org/package=dplyr).

72. R. Lenth, Emmeans: Estimated marginal means, aka least-squares means; https://CRAN.R-project.org/package=emmeans).

73. H. Wickham, *ggplot2: Elegant Graphics for Data Analysis* (Springer International Publishing, ed. 2, 2016).

74. D. Lüdecke, ggeffects: Tidy data frames of marginal effects from regression models. *J. Open Source Softw.* **3**, 772 (2018).

75. D. Bates, M. Mächler, B. Bolker, S. Walker, Fitting linear mixed-effects models **Using lme4**. *J. Stat. Soft.* **67**, 1–48 (2015).

76. A. Kuznetsova, P. B. Brockhoff, R. H. B. Christensen, lmerTest Package: Tests in linear mixed effects models. *J. Stat. Soft.* **82**, 1–26 (2017).

77. K. Bartoń, MuMIn: Multi-model inference (2022); https://CRAN.R-project.org/package=MuMIn.

78. J. Pinheiro, D. Bates, S. DebRoy, D. Sarkar, R Core Team, nlme: Linear and nonlinear mixed effects models (2022); https://CRAN.R-project.org/package=nlme.

79. G. E. Hoffman, E. E. Schadt, variancePartition: Interpreting drivers of variation in complex gene expression studies. *BMC Bioinformatics* **17**, 483 (2016).

80. S. Nakagawa, H. Schielzeth, A general and simple method for obtaining *R2* from generalized linear mixed-effects models. *Methods Ecol Evol.* **4**, 133–142 (2013).
